# Opinion: What defines high-risk CLL in the post-chemoimmunotherapy era?

**DOI:** 10.3389/fonc.2023.1106579

**Published:** 2023-02-09

**Authors:** Jennifer Edelmann, Jitka Malcikova, John C. Riches

**Affiliations:** ^1^ ClinSciNet - The Clinician Scientist Network, Münsingen, Germany; ^2^ Department of Internal Medicine – Hematology and Oncology, University Hospital Brno, Masaryk University, Brno, Czechia; ^3^ Center of Molecular Medicine, Central European Institute of Technology, Masaryk University, Brno, Czechia; ^4^ Centre for Haemato-Oncology, Barts Cancer Institute, Queen Mary University of London, London, United Kingdom; ^5^ Department of Haemato-Oncology, Barts Health NHS Trust, St. Bartholomew’s Hospital, London, United Kingdom

**Keywords:** chronic lymphocytic leukemia (CLL), high-risk, TP53, definition, BTK - Bruton’s tyrosine kinase, BCL2 (B-cell lymphoma 2), COVID - 19, risk factor

The definition of high-risk chronic lymphocytic leukemia (CLL) was relatively simple in the chemoimmunotherapy era, as it was defined by only one genomic marker, *TP53* alteration, along with poor responses to purine-analogue based treatment (1). While other biomarkers such as unmutated IGHV, del(11q), high ZAP70 expression and high CD38 expression were associated with inferior prognosis, *TP53* deficiency by mutation and/or del(17p) remained the only biomarker that clearly guided treatment decisions (2).

The emergence of targeted compounds has rendered chemoimmunotherapy virtually obsolete for CLL treatment, with it remaining an option only for patients with a mutated IGHV, normal *TP53* and a non-complex karyotype (3). Instead, non-chemotherapeutic targeted treatment has now become the standard of care. Approved treatment options in first- and second-line include continuous treatment with a covalent BTK inhibitor (e.g. ibrutinib, acalabrutinib) plus/minus anti-CD20 monoclonal antibody (4–9), fixed duration therapy with the BCL2 inhibitor venetoclax plus anti-CD20 monoclonal antibody (10, 11), fixed duration therapy with venetoclax plus ibrutinib (12, 13), and for *TP53* altered cases, continuous monotherapy with venetoclax (14). Moreover, clinical trials are currently evaluating triple drug regimens that combine BTK and BCL2 inhibitors with anti-CD20 treatment (15–18). Looking forward, non-covalent BTK inhibitors (e.g. pirtobrutinib and nemtabrutinib) (19, 20), BTK degraders (e.g. NX-2127) (21), and second-generation BCL2 inhibitors (e.g. Lisaftoclax) (22) are promising alternatives in clinical development, along with immunotherapeutic approaches such as CAR T-cells and bispecific antibodies.

The paradigm shift from chemoimmunotherapy to targeted therapy and the ever-increasing number of treatment options has meant that defining high-risk CLL is less straight-forward. This is mainly because BTK and BCL2 inhibitors have been demonstrated to markedly improve progression-free and overall survival (PFS and OS) in *TP53*-deficient and IGHV unmutated CLL patients (4, 5, 7, 9, 10, 14, 23–25). Limited data from clinical trials evaluating ibrutinib first-line and acalabrutinib have even raised the possibility that BTK-inhibition may overcome the adverse effects of *TP53* deficiency (5, 6, 9, 26). Although results from a direct PFS comparison between *TP53* deficient and non-deficient cases are not available yet, data from the SEQUOIA and ALPINE trials testing the second-generation covalent BTK inhibitor zanubrutinib in first-line and in relapsed/refractory CLL further support this hypothesis (27, 28). In contrast, *TP53* alterations remained prognostic for shorter PFS in studies on ibrutinib treatment of relapsed/refractory CLL (29–31). This difference may be explained by a high prior treatment load in the relapsed/refractory population leading to a selection of adverse risk factors associated with *TP53* deficiency such as high karyotype complexity (32–34). Genomic characterization of sequential samples taken pre-ibrutinib treatment and at disease progression demonstrated that *TP53*-deficient subclones were not necessarily responsible for ibrutinib failure. For instance, several studies on the clonal dynamics of *BTK* mutation as a frequent resistance mechanism towards covalent BTK inhibitors have shown that at relapse, *BTK* mutation can evolve within a *TP53* wild-type subclone while the *TP53*-deficient subclone is eliminated or remains effectively controlled (35–37).

With regards to BCL2 inhibition, clinical trial data revealed that fixed-duration first-line treatment with venetoclax in combination with obinutuzumab could not completely overcome the adverse effects of *TP53* deficiency (10), with corresponding results after combination with ibrutinib pending. As data on continuous venetoclax first-line treatment and on venetoclax re-exposure is also currently lacking, it remains unclear as to what extent the impact of *TP53* deficiency relates to the mode of action and what relates to the treatment duration (time-limited versus continuous).

Results from PFS comparisons between CLL cases with mutated and unmutated IGHV status suggested that continuous ibrutinib and acalabrutinib monotherapy was able to abrogate the negative prognostic impact of unmutated IGHV in treatment-naïve and in relapsed/refractory CLL (4, 5, 26, 30). In treatment arms combining ibrutinib with rituximab or obinutuzumab, the PFS seems to be shorter in the IGHV unmutated than mutated subgroup, but direct comparisons are missing and results on the combination of acalabrutinib and obinutuzumab did not suggest a prognostic impact of the IGHV status (5–7, 9). With regards to venetoclax-based fixed-duration therapy, unmutated IGHV status retained prognostic significance and one can speculate that as IGHV unmutated patients achieved high response rates and MRD negativity, shorter PFS may reflect the more proliferative nature of IGHV unmutated CLL cells potentially leading to a faster re-growth of the CLL clone after end of treatment (10, 38–40).

Given the long PFS in IGHV unmutated (7-year PFS 58% in the RESONATE-2 trial) (4) and in *TP53* altered CLL cases (6-year PFS 61% in a phase II clinical trial) (23) that can already be achieved by continuous BTK inhibition in first-line, these characteristics should no longer be seen as high-risk features for treatment failure *per se*. They should rather be seen as factors associated with an increased risk for early disease progression in certain therapeutic regimens. To fully evaluate the impact of *TP53* alteration and IGHV status, longer follow-up data and more direct PFS comparisons of *TP53* altered versus non-altered and IGHV mutated versus unmutated cases are clearly required for all targeted treatment approaches. Likewise, disease and patient characteristics beyond *TP53* and IGHV must be validated or newly defined, and potentially integrated in new prognostic models, since risk scores like the CLL International Prognostic Index (CLL-IPI) and the Continuous Individualized Risk Index (CIRI)) were developed using data from patients treated by chemoimmunotherapy with re-evaluation in the context of novel agents pending (41, 42). For patients treated with ibrutinib, a four-factor scoring system involving *TP53* alterations, prior treatment, serum β2-microglobulin concentration, and lactate dehydrogenase level was developed to identify patients at increased risk of ibrutinib failure by the time of treatment initiation and relapse (43). This prognostic score is independently evaluated (44, 45), but remains to be evaluated in clinical trials testing second-generation covalent BTK inhibitors.

The absence of fully validated prospective biomarkers and generally valid risk scores stratifying treatment outcome has led to a return to a clinical definition of high-risk CLL: as being described by dual resistance towards BTK and BCL2 inhibition (46). While this approach can help to select patients for more perilous treatment strategies such as allogeneic stem cell transplantation, the obvious limitation is that this “post-hoc” definition comes too late for the patients. Hence, there remains a requirement to define biomarkers that identify high-risk disease at the time of diagnosis or first relapse.

Analyses of CLL cells resistant towards BTK or BCL2 inhibitors have identified biomarkers that predict for non-durable response to targeted treatments (33, 34, 47). Genomic instability is one example, possibly due to it facilitating the evolution of clones resistant to the selective pressure of therapy (33, 34). High levels of pro-proliferative stimuli driven by *MYC* gain, constitutive BCR-signaling and loss of cell-cycle control (e.g. by *CDKN2A*/*CDKN2B* deletion) may have similar effects on clonal evolution and drive CLL cells towards transformation (48–50). Furthermore, the immune microenvironment has been shown to play a crucial role in CLL, but it is not clear how to integrate these factors into risk stratification models (51).

Besides these non-treatment-specific risk factors, the acquisition of resistance mutations in *BTK*, *PCL2G* or *BCL2* represents an alternative mechanism of resisting the relevant inhibitor (52–56). While it is tempting to speculate that patients with these risk factors may benefit from treatment intensification with multi-agent combinations, prospective validation of this assumption is challenging as resistance mutations cannot be anticipated at the time of treatment initiation.

Therefore, the “brave new world of personalized CLL medicine” (51) remains a distant goal, with isolated analyses of putative biomarkers in individual clinical trial cohorts struggling to bring it closer. Biomarkers should be seen within the context of pathobiology and grouped for the definition of molecular CLL subtypes that will derive the most benefit from specific drug classes or treatment combinations (57). To reach that goal, collaborative initiatives as the CLL HARMONY Alliance are vital to compile patient registries that incorporate clinical trial as well as real-world data. This requires the application of complex “big data” analytical techniques including artificial intelligence and machine learning to identify the best biomarkers, to clearly define patient subgroups and to develop tailored therapeutic approaches.

Apart from this focus on the CLL cells and their biological heterogeneity, we feel that the definition of “high-risk CLL” should be broadened by including factors such as individual patient characteristics, treatment design, and the situational context of a patient’s care (see **Figure 1**). Some of these factors were already encapsulated within former CLL treatment algorithms such as the “go-go”, “slow-go” and “no-go” three-tier “traffic light” approach developed during the chemotherapy era (58). The enhanced tolerability of targeted therapies has led to this approach becoming less important since a wider range of patients can now benefit from highly effective treatments, but on the other hand, targeted therapies have brought a new set of considerations that impact outcome.

**Figure 1:**
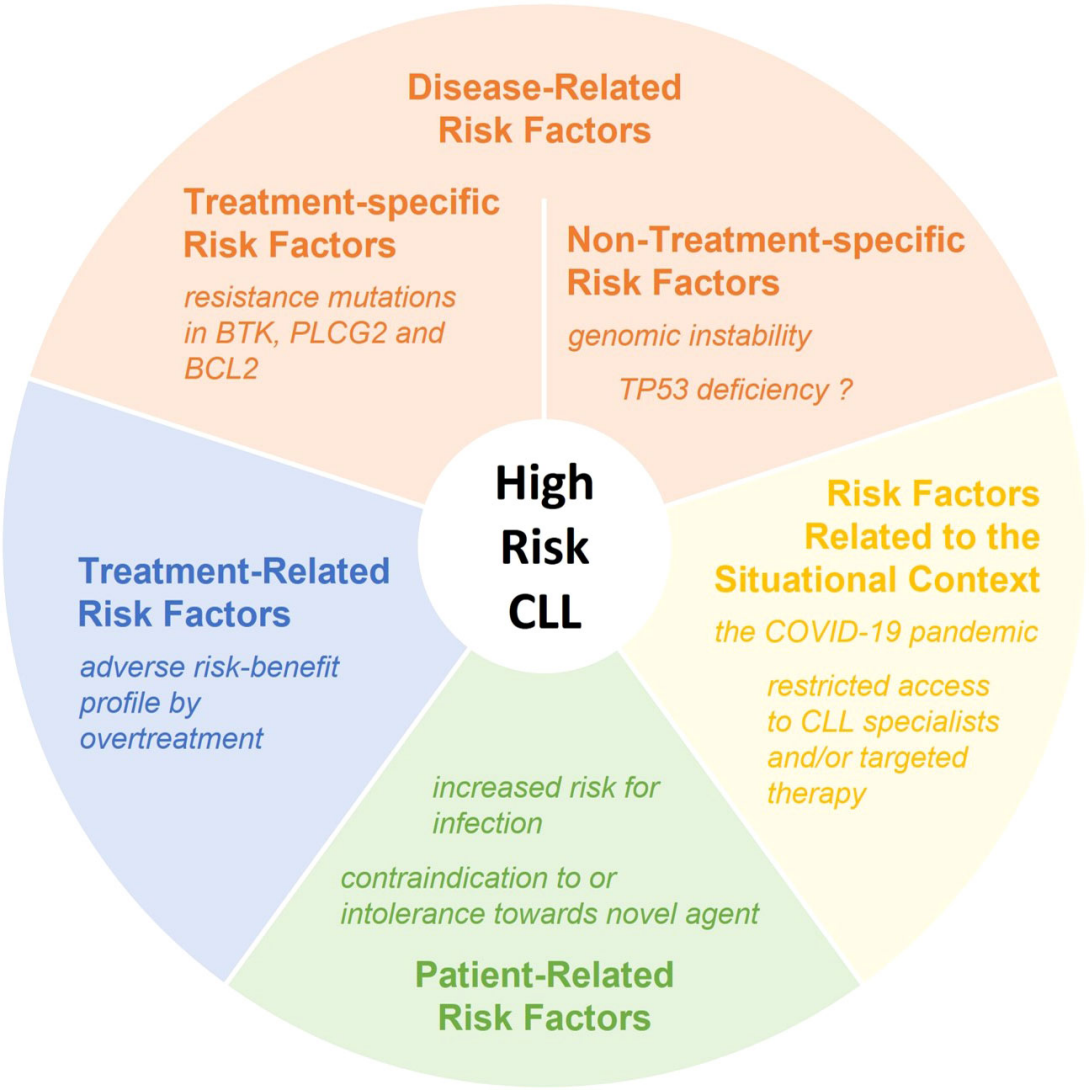
Factors increasing the risk for early CLL-related death. Examples are provided for each category of risk factors.

For example, the situational context of a patient can become a risk factor when access to CLL specialists is restricted or when the health care system of a country does not permit the prescription of more expensive targeted therapies. Moreover, patients who have a contraindication to or are intolerant towards one of the novel agents lack an important treatment option, which may become critical over the course of the disease. A patient with mechanical heart valve requiring anticoagulation could hence be regarded as high-risk due to BTK inhibition contraindication, even if high-risk biological factors are lacking. Another good example is the risk from infection, which has been brought into sharp focus in the context of the COVID-19 pandemic. Infection is a major cause of morbidity and mortality for CLL patients, including those with early-stage disease (59–61). This risk can be aggravated by treatment, as for instance, both anti-CD20 monoclonal antibodies and BTK inhibitors are associated with reduced ability to respond to anti-COVID-19 vaccination (62–64). Therefore, the pandemic has shown very clearly how the situational context can change a patient’s individual risk of harm from a certain treatment approach and that it remains important to balance the benefit and risks from treatment to avoid overtreatment. As an example, the benefit from addition of anti-CD20 therapy to targeted therapy must be critically evaluated particularly for BTK inhibitors, as the addition of rituximab to ibrutinib was shown to provide no clinical benefit (5, 31). Furthermore, the choice between a monotherapy, a dual therapy or a triple drug regimen must be adjusted to the patient’s individual risk profile to avoid situations where the risks of serious or even fatal adverse events from treatment exceed the risks from the disease itself.

Taken together, we believe that a more holistic definition of “high-risk CLL” would be to define it simply as any patient who has an increased risk of early CLL-related death. This could be from treatment, from infection, or many other factors on top of risks from the disease itself. With this definition in mind, risk assessment would be based on a combination of “prospective” biomarkers, such as *TP53* alterations, IGHV mutation status and karyotype complexity and “retrospective” factors, such as the duration of response to, and side effects from, a particular therapy. It would hence require regular updates over the disease course as suggested by the CIRI score (42). Such perspective would encourage investigators conducting future clinical trials to focus on the elements influencing overall survival, with greater consideration of a patient’s journey through multiple lines of treatment rather than just a single intervention. This would be stark contrast to the current situation where, for example, patients with a contraindication to one drug class are excluded from the relevant clinical trial. While a prospective approach would be the ideal, this will be close to impossible due to the timescales and rapid evolution of therapies. Alternatively, large-scale retrospective analyses could be employed to determine the best sequencing of drugs across multiple treatment lines for molecularly and/or risk stratified patient subgroups. Future research should therefore aim to incorporate all of the elements described above to tailor treatment towards the specific circumstances of individual patients.

## Author contributions

All authors listed above have made a substantial, direct and intellectual contribution to the work, wrote the article and approved it for publication.
